# Functional labeling of individualized postsynaptic neurons using optogenetics and *trans-*Tango in *Drosophila* (FLIPSOT)

**DOI:** 10.1371/journal.pgen.1011190

**Published:** 2024-03-14

**Authors:** Allison N. Castaneda, Ainul Huda, Iona B. M. Whitaker, Julianne E. Reilly, Grace S. Shelby, Hua Bai, Lina Ni

**Affiliations:** School of Neuroscience, Virginia Polytechnic Institute and State University, Blacksburg, Virginia, United States of America; Instituto Leloir, ARGENTINA

## Abstract

A population of neurons interconnected by synapses constitutes a neural circuit, which performs specific functions upon activation. It is essential to identify both anatomical and functional entities of neural circuits to comprehend the components and processes necessary for healthy brain function and the changes that characterize brain disorders. To date, few methods are available to study these two aspects of a neural circuit simultaneously. In this study, we developed FLIPSOT, or functional labeling of individualized postsynaptic neurons using optogenetics and *trans-*Tango. FLIPSOT uses (1) *trans-*Tango to access postsynaptic neurons genetically, (2) optogenetic approaches to activate (FLIPSOTa) or inhibit (FLIPSOTi) postsynaptic neurons in a random and sparse manner, and (3) fluorescence markers tagged with optogenetic genes to visualize these neurons. Therefore, FLIPSOT allows using a presynaptic driver to identify the behavioral function of individual postsynaptic neurons. It is readily applied to identify functions of individual postsynaptic neurons and has the potential to be adapted for use in mammalian circuits.

## Author summary

The human brain, like that of other animals, is composed of neurons. These neurons receive and integrate information from external environments and determine output behaviors. To understand the brain, it is not only necessary to know the neurons that make up the brain and how they are connected, but also to understand the function of each individual neuron. To better control and treat brain disorders, we also need to compare the differences in neuronal connections and functions between healthy and unhealthy brains. As of now, there are few methods that allow us to simultaneously observe both the connections and functions of neurons. Here, we developed a genetic tool called FLIPSOT, which stands for functional labeling of individualized postsynaptic neurons using optogenetics and *trans-*Tango. FLIPSOT not only allows us to see which neurons are connected to the ones we are interested in, but also can activate or inhibit one or several of these neurons, enabling us to understand their functions. Currently, this technology is only used in fruit flies. We hope it can be applied to mammals in the near future.

## Introduction

A population of neurons that are interconnected by synapses constitutes a neural circuit, which performs specific functions upon activation [1]. Neural circuits are the fundamental framework for all brain activities, such as processing perception and cognition and coordinating behavior. Impairments in neural circuits can lead to a wide range of neurodegenerative and psychiatric disorders [2–4]. Therefore, it is essential to identify both anatomical and functional entities of neural circuits to comprehend the components and processes necessary for healthy brain function and the changes that characterize brain disorders.

*Drosophila melanogaster* is an attractive model to study the circuit basis of animal behavior because of its relatively small nervous system, sophisticated and robust behaviors, and extensive collections of genetic reagents to label and manipulate various classes of neurons [5]. Multiple techniques have been developed to investigate *Drosophila* neural circuits, including serial-section electron microscopy (EM) [6–8], paired-recording [9,10], photoactivatable GFP (PA-GFP) [11–17], and calcium imaging [6,18]. Each method has its own advantage. EM and PA-GFP can indicate synaptic connections and identify the morphology of potential postsynaptic neurons. Calcium imaging and paired recording provide information on functional connections between neurons. However, none of these techniques simultaneously track the synaptic connections and evaluate the functional relevance of individual neurons in a circuit.

GFP Reconstitution Across Synaptic Partners (GRASP) is a well-established genetic transsynaptic tool labeling synapses based on the proximity of pre- and postsynaptic plasma membranes. GRASP allows for visualization of synaptic connection and activity between two neurons by light microscopy but requires driver lines of both pre- and postsynaptic neurons [19–27]. Recently, new genetic transsynaptic tools, including *trans*-Tango [28], TRACT (TRAnsneuronal Control of Transcription) [29], BAcTrace (Botulinum-Activated Tracer) [30] and *retro-*Tango [31], have been developed. Unlike GRASP, these methods rely on only one driver to label and provide genetic access to synaptic partners. Among them, *trans*-Tango is the most widely used tool for labeling synaptic partners in various *Drosophila* neural circuits [32]. The design of *trans-*Tango is based on the Tango assay, which converts transient interaction between G protein-coupled receptors (GPCRs) and their ligands to a more stable readout [28,33]. The GAL4 drives the expression of the *trans-*Tango ligand in presynaptic neurons, which binds to and activates the *trans-*Tango receptor (a GPCR) on postsynaptic membranes. The activated *trans-*Tango receptor, in turn, recruits β-arrestin2 linked to a protease. The protease releases QF from the *trans-*Tango receptor. QF then translocates to nuclei and expresses a reporter to visualize postsynaptic neurons. While optogenetic and calcium imaging techniques have been combined with *trans-*Tango to investigate the functional connectivity between pre- and postsynaptic neurons [34–37], these approaches cannot determine the behavioral importance of individual postsynaptic neurons.

In this study, we developed a tool named FLIPSOT or functional labeling of individualized postsynaptic neurons using optogenetics and *trans-*Tango. FLIPSOT allows the use of a presynaptic driver to identify the behavioral function of individual postsynaptic neurons. The heating cell (HC)-controlled circuit was used as a model to test our newly developed tool. HCs are peripheral warm receptors located in the arista of each antenna and are activated at about 25°C [38,39]. The HC-controlled circuit guides animals to avoid warm temperatures rapidly when exposed to a steep gradient [38,40,41]. To define the behavioral function of the postsynaptic neurons of HCs, single-fly thermotactic and optogenetic assays were set up. Optogenetic genes tagged with fluorescence markers were inserted downstream of an FRT-stop-FRT cassette to modulate neuronal activity in a random and sparse manner. We then combined *UAS-trans-Tango*, *hs-FLP*, *QUAS-mtdTomato-HA*, and *QUAS-FRT-stop-FRT-GtACR2*.*EYFP* (*FLIPSOTi*) to identify HC PNs necessary for rapid warm avoidance. Similarly, *UAS-trans-Tango*, *hs-FLP*, *QUAS-mCD8-GFP*, and *QUAS-FRT-stop-FRT-CsChrimson*.*mCherry* were combined to create *FLIPSOTa* to determine HC PNs sufficient for avoidance. FLIPSOT is readily applied to identify functions of individual postsynaptic neurons in many other fruit fly neural circuits and has the potential to be adapted for use in mammalian circuits.

## Results

### The design of FLIPSOT

**Fig 1:**
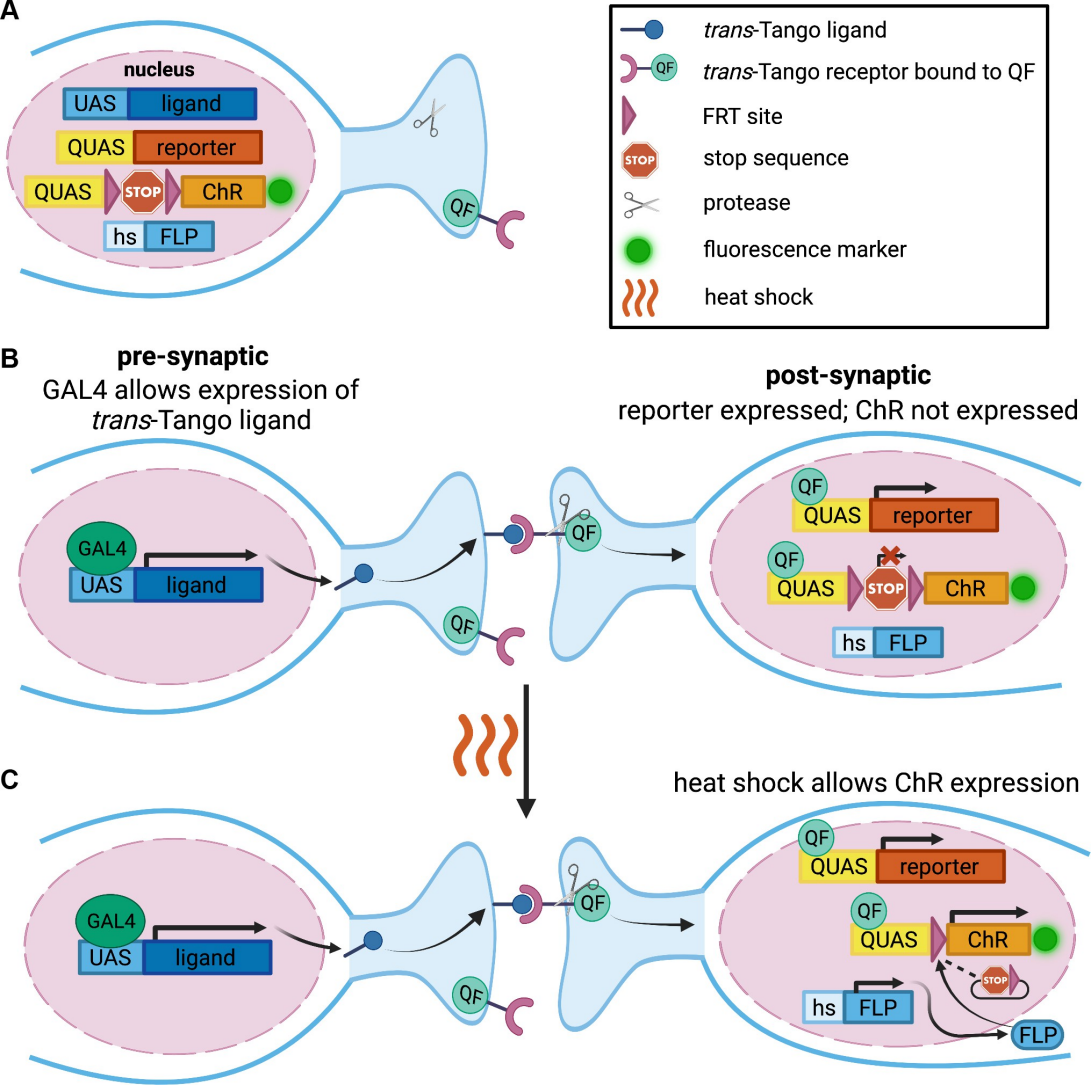
Schematic design of FLIPSOT. (A) Genetic components in FLIPSOT include a *trans-*Tango ligand, *trans-*Tango receptor bound to QF, protease, FLP, fluorescence reporter, and optogenetic tool (ChR) tagged with a fluorescence marker. The *trans-*Tango ligand is downstream of a UAS sequence, a binding site of the transcription activator GAL4. Both the *trans-*Tango receptor and protease are pan-neuronally expressed. When the ligand binds to the receptor on synaptically connected neurons, the protease is recruited to release the transcription activator QF from the *trans-*Tango receptor in postsynaptic neurons. The reporter is downstream of a QUAS sequence, a binding site of QF. Heat shock permits the expression of FLP. The optogenetic gene is under the control of QUAS and an FRT-stop-FRT cassette, so both QF and FLP must translocate to nuclei to allow its expression, which is visualized by a tagged fluorescence protein. (B,C) A presynaptic GAL4 drives the expression of the *trans-*Tango ligand in presynaptic neurons. The ligand binds to the *trans-*Tango receptor on synaptically connected neurons and releases QF. QF translocates to nuclei to express the reporter. Although QF binds to the QUAS sequence upstream of the optogenetic gene, the optogenetic gene will not be expressed (B) until heat shock allows the expression of FLP, which removes the stop sequence from the FRT-stop-FRT cassette randomly to permit its expression (C). This figure was created with BioRender.com.

To generate a genetic tool that uses a presynaptic driver to label and manipulate postsynaptic neurons randomly and sparsely, we incorporate the following genetic components in FLIPSOT (Fig 1A). The *trans*-Tango allows for the genetic access of postsynaptic neurons by a presynaptic driver [28]. The *QUAS-reporter*, controlled by *trans-*Tango, enables the visualization of postsynaptic neurons (Fig 1B). The channelrhodopsins (ChRs), CsChrimson [42] and GtACR2 [43–45], were used to manipulate the activity of postsynaptic neurons. CsChrimson is activated by red light to depolarize cells. GtACR2 is activated by blue light to hyperpolarize cells. Fluorescence markers (mCherry and EYFP) are tagged to ChRs to visualize postsynaptic neurons expressing ChRs. ChRs are downstream of a QUAS sequence to enable *trans-*Tango to control the expression of postsynaptic ChRs. An FRT-stop-FRT cassette is inserted between QUAS and ChRs [46]. The stop sequence blocks ChR expression without a FLP recombinase [47]. When heat shock is applied, FLP removes the stop sequence in some, but not all, postsynaptic neurons, allowing random and sparse postsynaptic neurons to express ChRs and their fluorescence markers Fig 1C). Single-fly behavioral assays are required to pinpoint the function of postsynaptic neurons. In total, FLIPSOT includes *UAS-trans-Tango*, *hs-FLP*, and *QUAS-FRT-stop-FRT* followed by a fluorescently tagged ChR. We also include a reporter under QUAS control to serve as an internal control labeling all postsynaptic neurons. A presynaptic GAL4 line is necessary to drive FLIPSOT. The setup of single-fly behavioral assays and the development of the individual genetic components of the FLIPSOT tool are described in following sections.

### Set up single-fly thermotactic and optogenetic assays

**Fig 2:**
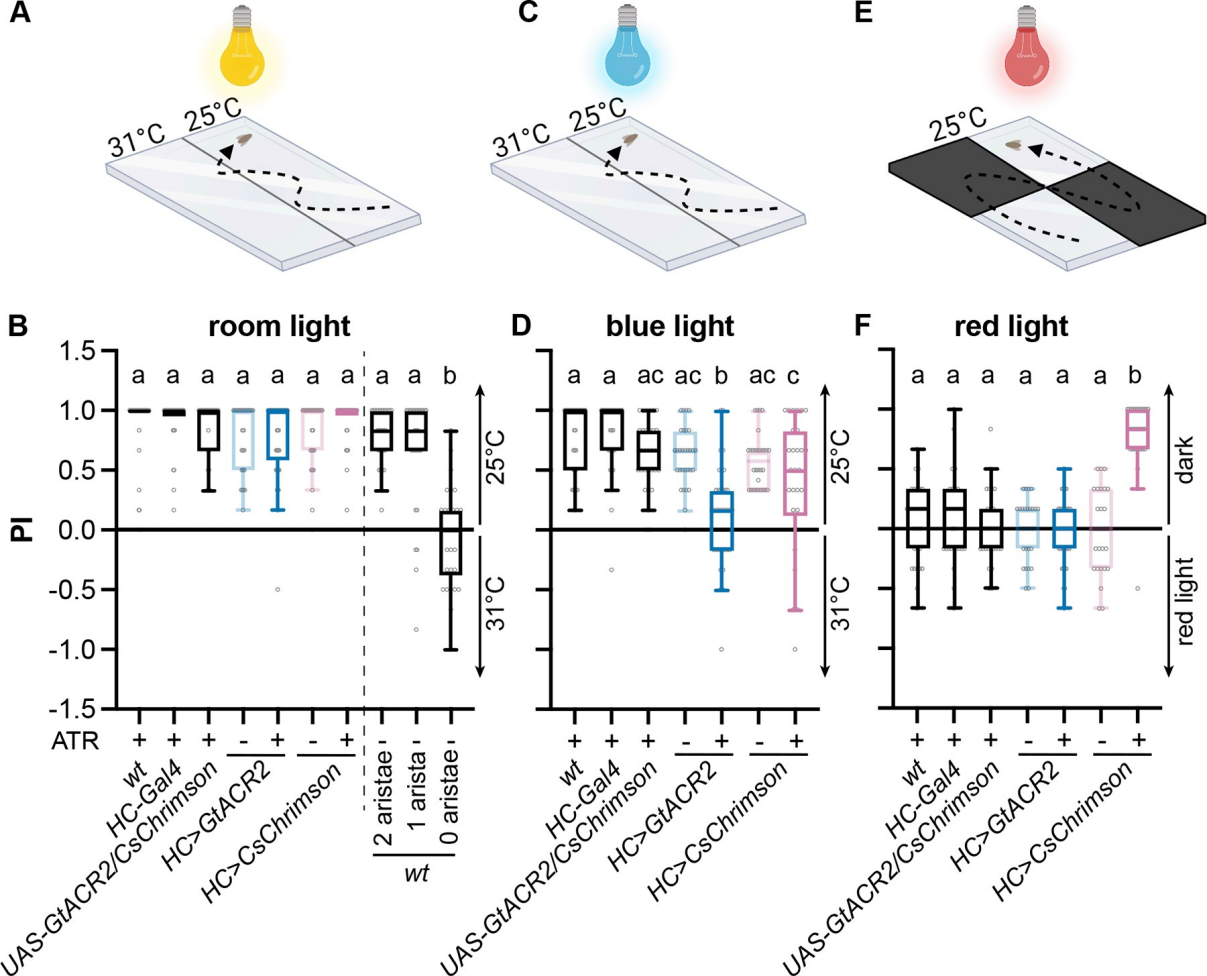
Single-fly thermotactic and optogenetic assays. (A) Setup for the single-fly two-choice thermotactic assay under ambient light condition. Flies were given one minute to choose between 25 and 31°C (created with BioRender.com). (B) PI of the indicated genotypes that were raised with (+) or without (-) dietary retinal (ATR). *n* = 30–45. Boxes are defined by 25^th^ to 75^th^ percentiles; internal lines show median; whiskers extend 1.5 times interquartile range. Kruskal-Wallis test followed by Dunn’s multiple comparisons test; letters denote statistically distinct groups, *p* < 0.05. Genotypes: *UAS-GtACR/CsChrimson* is *UAS-GtACR2*.*EYFP/UAS-CsChrimson*.*mVenus*; *HC>GtACR2* is *HC-Gal4;UAS-GtACR2*.*EYFP; HC>CsChrimson* is *HC-Gal4;UAS-CsChrimson*.*mVenus*. (C) Setup for the single-fly two-choice thermotactic assay under blue light condition. Flies were given one minute to choose between 25 and 31°C (created with BioRender.com). (D) PI of the indicated genotypes that were raised with (+) or without (-) dietary retinal (ATR). *n* = 30–45; Kruskal-Wallis test followed by Dunn’s multiple comparisons test; letters denote statistically distinct groups, *p* < 0.05. (E) Setup for the single-fly optogenetic assay under red light condition. Flies were given one minute to choose between the dark and light environment (created with BioRender.com). (F) PI of the indicated genotypes that were raised with (+) or without (-) dietary retinal (ATR). *n* = 30–45; Kruskal-Wallis test followed by Dunn’s multiple comparisons test; letters denote statistically distinct groups, *p* < 0.05. See also S1 Fig.

Because of the inherent stochastic nature of FLIPSOT, single-fly behavioral assays are required to analyze the behavioral roles of individual postsynaptic neurons. In this study we used the HC-controlled thermosensory circuit as a model to develop FLIPSOT. HCs guide rapid warm avoidance, and thus we first implemented a single-fly rapid warm avoidance assay to assess whether this circuit is an appropriate model (Fig 2A). Briefly, a single fly was acclimated under a transparent cover (2 mm (height) X 58 mm (width) X 83 mm (length)) for 15–25 seconds. Then, it was allowed to choose between 25°C and 31°C for one minute. The preference index (PI) was calculated as the ratio of the difference between the time the fly spent at 25°C and 31°C to the total time. All tested genotypes with intact aristae avoided 31°C (Fig 2B). Neither fly genders nor the exchange of temperature sides affected warm avoidance (S1 Fig). Importantly, *wild-type* flies with both aristae ablated did not exhibit a preference between 25 and 31°C, while flies with a single arista ablated avoided 31°C (Fig 2B).

Then, we set up single-fly optogenetic assays to test whether GtACR2 and CsChrimson could suppress and activate the neural circuit controlling rapid warm avoidance, respectively. Either GtACR2 or CsChrimson was expressed in HCs by *HC-Gal4*. A blue light was used to replace the regular ambient room light to activate GtACR2 (Fig 2C). Similarly, a single fly was acclimated for 15–25 seconds before being given a choice between 25 and 31°C for one minute under blue light. Flies expressing GtACR2 in HCs failed to avoid 31°C, suggesting HCs are necessary for rapid warm avoidance (Fig 2D).

A red light was used to activate CsChrimson (Fig 2E). In this assay, HC activation did not depend on the temperature increase. Instead, they were activated by red light due to their expression of CsChrimson, and thus, flies were not exposed to different temperatures. The cover was created with two opposing transparent quadrants and two opaque quadrants. The red light illuminated the cover from above. When flies passed the transparent quadrants, CsChrimson was activated by red light. The fly was given one min to choose between dark and red-light quadrants. Control flies did not show a preference between dark and red-light quadrants. However, flies expressing CsChrimson in HCs significantly preferred dark quadrants, demonstrating that activation of HCs is sufficient to drive avoidance of red light (Fig 2F).

### HCs connect to four tracts of PNs

**Fig 3:**
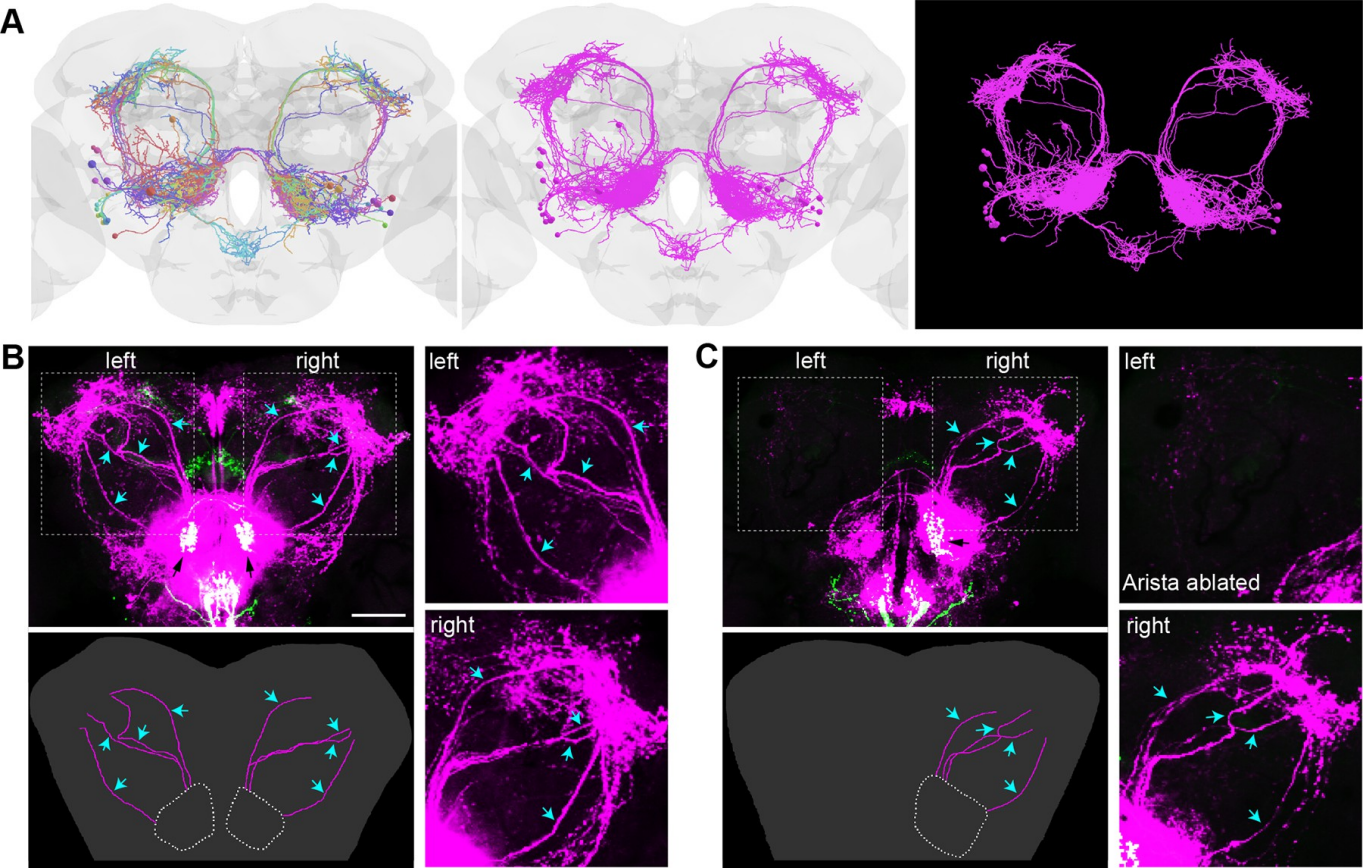
HCs connect to four tracts of PNs. (A) The full adult fly brain EM data revealed 21 PNs relaying information from VP2. Left, each neuron in a unique color; middle, all PNs in magenta; right, same as the middle panel with the brain structure removed. The brain model was generated using Virtual Fly Brain database and visualized using opensource packages in python. (B) In flies bearing *trans*-Tango components, driving ligands and GFP expressing in VP2 (black arrows) of the AL by *HC-Gal4* results in mtdTomato expression (magenta) in postsynaptic LNs and PNs (cyan arrows). Scale bar: 50 μm. Left lower panel: illustration of PNs; right upper panel: left PNs; right lower panel: right PNs. (C) The ablation of the left arista results in the absence of the left PNs while leaving the right PNs (cyan arrows) unaffected. Left lower panel: illustration of PNs; right upper panel: left PNs; right lower panel: right PNs. See also S2 Fig.

HCs project their axonal termini to VP2, a glomerulus of the antennal lobe (AL) in the brain [48,49]. The postsynaptic neurons of HCs consist of local interneurons (LNs) and projection neurons (PNs) that are the output cells of the AL. EM tracing has identified four tracts of projection neurons (PNs) from VP2: a medial AL tract (mALT), a mediolateral AL tract (mlALT), a transversal AL tract (tALT), and a lateral AL tract (IALT) [50,51]. The mALT comprises at least 14 PNs, while lALT includes five PNs, and mlALT and tALT each contain one PN [50] (Fig 3A).

FLIPSOT relies on *trans-*Tango to access postsynaptic neurons. We thus used *HC-Gal4* to drive *trans-*Tango and examined whether postsynaptic neurons of HCs were properly labeled. Projections of *HC-Gal4-*positive neurons in the brain were observed in the VP2 glomerulus of the antennal lobe (AL), the subesophageal zone (SEZ), and the fan-shaped body (FB) (Fig 3B). Besides local interneurons (LNs) in the AL and SEZ, four tracts of PNs from the AL, mALT, mlALT, tALT, and lALT, exhibited a strikingly similar pattern as observed in EM tracing. These PNs were arborated in the mushroom body (MB), lateral horn (LH), and posterior lateral protocerebrum (PLP). Postsynaptic neurons were also observed in the superior medial protocerebrum (SMP). GR28B(D) is the warm sensor in HCs [38]. Flies using *Gr28b*.*d-Gal4* to drive *trans-*Tango exhibited similar patterns of pre- and postsynaptic neurons (S2 Fig).

The ablation of one arista diminished the projections of *HC-Gal4* positive neurons in VP2 but did not affect those in the SEZ and FB (Fig 3C). In the AL, the mtdTomato expression was decreased significantly on the side the arista was removed, and PNs were not detectable. However, the postsynaptic labeling in the SEZ and SMP was not affected. These findings suggested that HCs project to VP2 and synaptically connect to PNs and LNs in the AL. Since PNs send information to higher-level brain regions, along with the behavioral analysis of flies with one arista ablated (Fig 2B), we concluded that single-side PNs are sufficient for guiding warm avoidance behavior.

### Random and sparse labeling

FLIPSOT requires random and sparse labeling and modulating postsynaptic neurons. We applied the FRT-stop-FRT cassette to achieve the stochasticity of FLIPSOT and included optogenetic tools, CsChrimson and GtACR2, to activate or inhibit postsynaptic neurons. Therefore, we created two transgenic fly lines: *QUAS-FRT-stop-FRT-GtACR2*.*EYFP* and *QUAS-FRT-stop-FRT-CsChrimson*.*mCherry*. GtACR2 and CsChrimson were cloned downstream of QUAS to allow *trans-*Tango to control their expression. An FRT-stop-FRT cassette was inserted between QUAS and optogenetic genes. Fluorescence tags, EYFP and mCherry, were added at the C termini of optogenetic genes to enable the visualization of the neurons expressing GtACR2 and CsChrimson, respectively.

**Fig 4:**
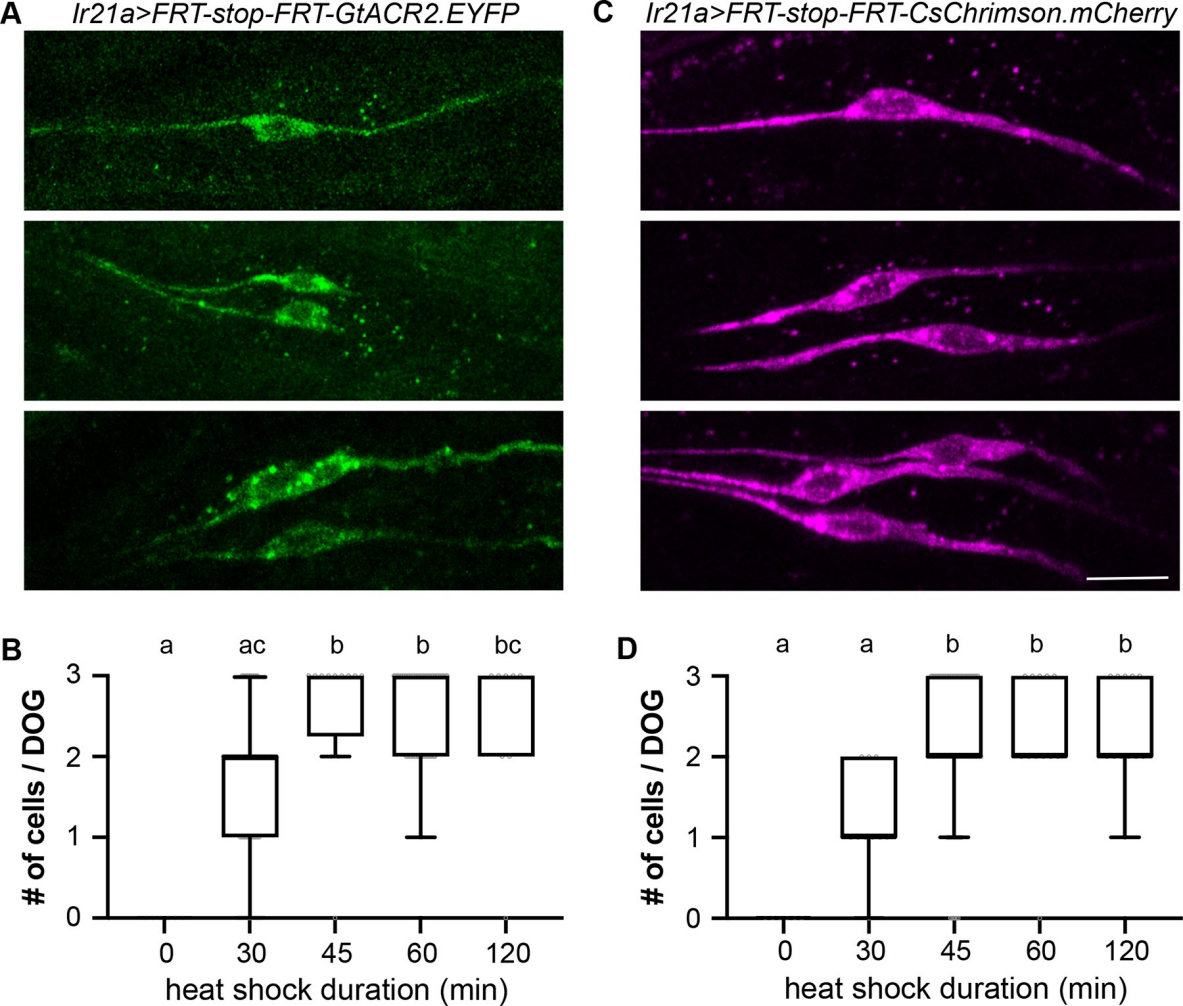
Heat shock removes the stop sequence from the FRT-stop-FRT cassette randomly. (A) After heat shock, each DOG may contain zero, one (upper), two (middle), or three (lower) GtACR2.YFP-positive *Ir21a-QF2w-*labeled neurons. *Ir21a>FRT-stop-FRT-GtACR2*.*EYFP* is *hs-FLP;Ir21a-QF2w/QUAS-FRT-stop-FRT-GtACR2*.*EYFP*. Scale bar: 10 μm. (B) The number of GtACR2.YFP-positive *Ir21a-QF2w-*labeled neurons in each DOG after incubating larvae at 35°C for the indicated periods. *N* = 6–62; Kruskal-Wallis test followed by Dunn’s multiple comparisons test; letters denote statistically distinct groups, *p* < 0.05. (C) After heat shock, each DOG may contain zero, one (upper), two (middle), or three (lower) CsChrimson.mCherry-positive *Ir21a-QF2w-*labeled neurons. *Ir21a>FRT-stop-FRT-CsChrimson*.*mCherry* is *hs-FLP;Ir21a-QF2w/QUAS-FRT-stop-FRT-CsChrimson*.*mCherry*. Scale bar: 10 μm. (D) The number of CsChrimson.mCherry-positive *Ir21a-QF2w-*labeled neurons in each DOG after incubating larvae at 35°C for the indicated periods. *n* = 6–80; Kruskal-Wallis test followed by Dunn’s multiple comparisons test; letters denote statistically distinct groups, *p* < 0.05. See also S3 and S4 Figs.

To assess the stochastic removal of the stop sequence in *QUAS-FRT-stop-FRT-GtACR2*.*EYFP* and *QUAS-FRT-stop-FRT-CsChrimson*.*mCherry*, and the subsequent labeling of cells by EYFP and mCherry, we employed a readily quantifiable set of neurons. We used *Ir21a-QF2w* to drive *QUAS-FRT-stop-FRT-GtACR2*.*EYFP* and *QUAS-FRT-stop-FRT-CsChrimson*.*mCherry* and expressed FLP using a heat shock promoter (*hs-FLP*). *Ir21a-QF2w* labels three neurons in each dorsal organ ganglion (DOG) in fly larvae (S3 Fig). Heat shock allowed for FLP expression. In *Ir21a-QF2w* positive cells, the stop sequence was removed, and GtACR2.EYFP or CsChrimson.mCherry was expressed (Fig 4A and 4C). Since FLP was inadequate to trigger recombination in all cells, each DOG might have zero, one, two, or three cells expressing EYFP or mCherry (Fig 4A and 4C). The expression ratio of fluorescence markers and heat shock duration were correlated. Elongation of heat shock increased the expression ratio (Fig 4B and 4D). Heat shock also randomly removed the stop sequence between two FRT sites in adult HCs (S4 Fig). These results suggested that managing heat shock duration enables the random and sparse expression of GtACR2.EYFP or CsChrimson.mCherry. In the following experiments, we applied 30 min heat shock to achieve the sparse expression of GtACR2.EYFP and CsChrimson.mCherry.

### Necessary and sufficient PNs for rapid warm avoidance

Finally, we combined *UAS-trans-Tango, hs-FLP, QUAS-mtdTomato-HA*, and *QUAS-FRT- stop-FRT-GtACR2.EYFP* to generate *FLIPSOTi*. Similarly, *UAS-trans-Tango, hs-FLP, QUAS-mCD8-GFP*, and *QUAS-FRT-stop-FRT-CsChrimson.mCherry* were combined to create *FLIPSOTa*. *FLIPSOTi* and *FLIPSOTa* were designed to determine individual postsynaptic neurons that were necessary and sufficient, respectively, to transduce information for the behaviors controlled by presynaptic neurons.

**Fig 5:**
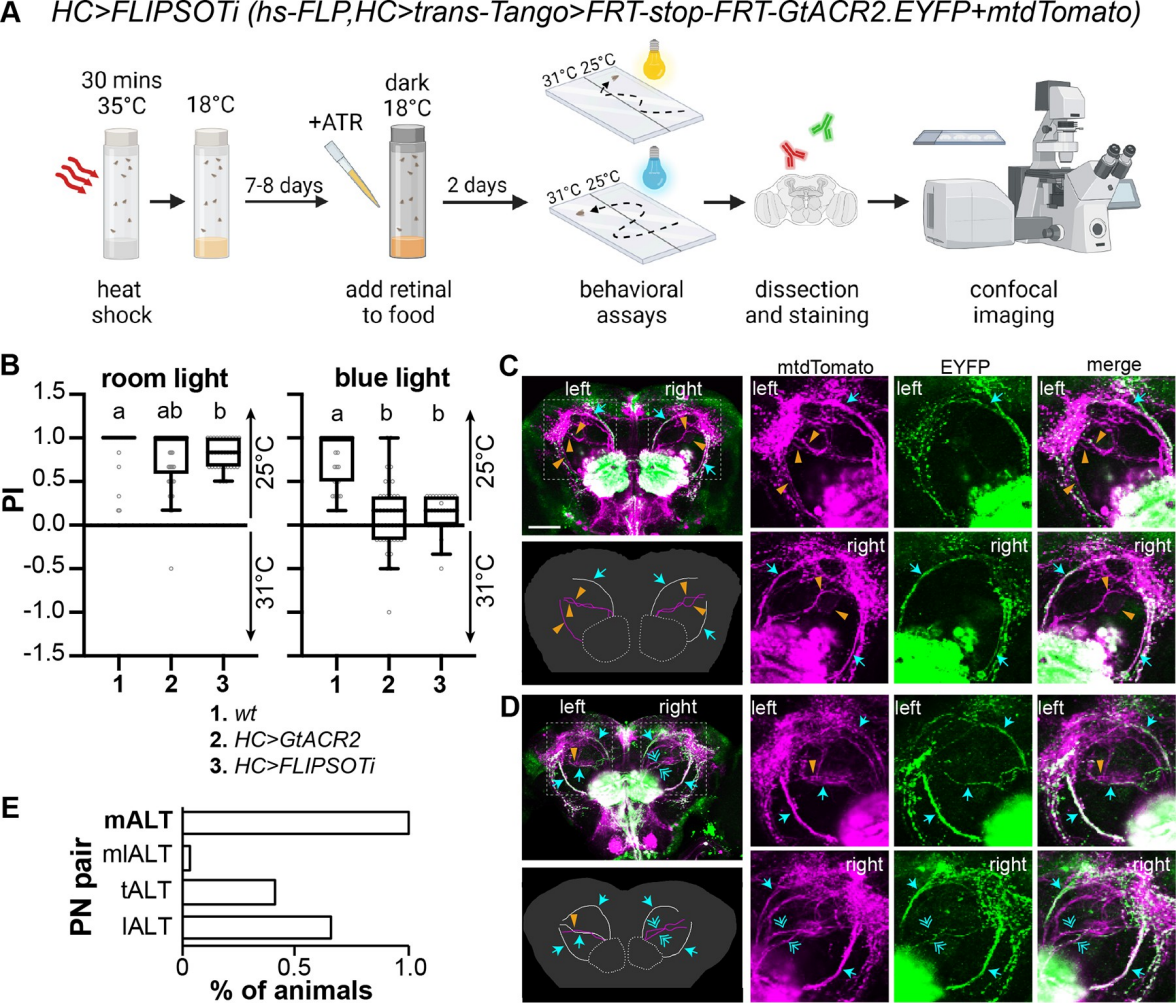
Identify PNs necessary to drive rapid warm avoidance. (A) Schematic representation of identifying PNs necessary for the avoidance behavior. *FLISOTi* is *hs-FLP;UAS-trans-Tango/QUAS-mtdTomato;QUAS-FRT-stop-FRT-GtACR2*.*EYFP*. The pre-synaptic driver is *HC-Gal4* (created with BioRender.com). (B) PI of the indicated genotypes to avoid 31°C under ambient room (left) or blue (right) light. Flies were raised with dietary retinal (ATR) in the dark for two days before experiments. *n* = 31–45; Kruskal-Wallis test followed by Dunn’s multiple comparisons test; letters denote statistically distinct groups, *p* < 0.05. The behavioral datasets of *wt* and *HC>GtACR2* (*HC-Gal4;UAS-GtACR2*.*EYFP)* from Fig 1B and 1D are included for comparison with *HC>FLIPSOTi* flies. (C,D) GtACR2.EYFP-positive HC PNs in flies not avoiding 31°C under blue light. Left upper panels: immunohistochemistry. Magenta: HA antibody to show all postsynaptic neurons of HCs, green: YFP antibody to indicate GtACR2.EYFP-positive postsynaptic neurons of HCs; cyan arrow: GtACR2.EYFP-positive PN, orange arrowhead: GtACR2.EYFP-negative PN; cyan double arrow: GtACR2.EYFP-weak PN; if the EYFP staining is much weaker than the HA staining, the PN is considered to lack GtACR2.EYFP. Scale bar: 50 μm. Left lower panels: illustration of PNs. White: GtACR2.EYFP-positive PN, magenta: GtACR2.EYFP-negative PN. Right three upper panels: left PNs; right three lower panels: right PNs. (C) Three PN tracts express GtACR2.EYFP (both mALTs and the right lALT). (D) Five PNs express GtACR2.EYFP (left: mALT, tALT, and lALT; right: mALT and lALT). (E) The mALT PNs are necessary for rapid warm avoidance. We analyzed 29 brains, all of which expressed GtACR2.EYFP-positive mALT at both sides. See also S5 and S6 Figs.

HCs are necessary and sufficient to guide rapid warm avoidance [38,41,52]. We first used *HC-Gal4* to express *FLIPSOTi* to identify individual PNs necessary for rapid warm avoidance (Fig 5A). Newly eclosed flies were exposed to 35°C for 30 min and then kept at 18°C. While the original report suggests that *trans*-Tango development requires incubating flies at 18°C for two to three weeks [28], a recent study indicates that achieving optimal expression also depends on QUAS fly lines [37]. For *QUAS-FRT-stop-FRT-GtACR2.EYFP*, incubation at 18°C for ten days achieved the optimal expression. Food was supplemented with *ATR* two days before behavioral tests. Single-fly rapid warm avoidance assays were conducted under regular ambient room light and blue light. Among 117 flies that had normal avoidance in room light, 29 flies exhibited diminished avoidance in blue light and were collected for brain dissection (Figs 5B and S5A). Fly brains were dissected, and PNs expressing GtACR2.EYFP were identified. In FLIPSOTi, *QUAS-mtdTomato-HA* was used as a control to visualize postsynaptic neurons. Four tracts of PNs were observed on each hemisphere (magenta). In Fig 5C, the mALT was the only tract expressing GtACR2.EYFP (green) in the left brain hemisphere, while both mALT and lALT expressed GtACR2.EYFP in the right brain hemisphere. In Fig 5D, the left mlALT did not express GtACR2.EYFP. In the right brain hemisphere, all four tracts were visible by EYFP. However, EYFP signals in the mlALT and tALT were faint compared to mtdTomato, and thus the mlALT and tALT were considered to lack GtACR2.EYFP. All 29 brains expressed the mALT on both sides (Figs 5E and S6), suggesting the mALT from VP2 is necessary to transduce information for rapid warm avoidance.

**Fig 6:**
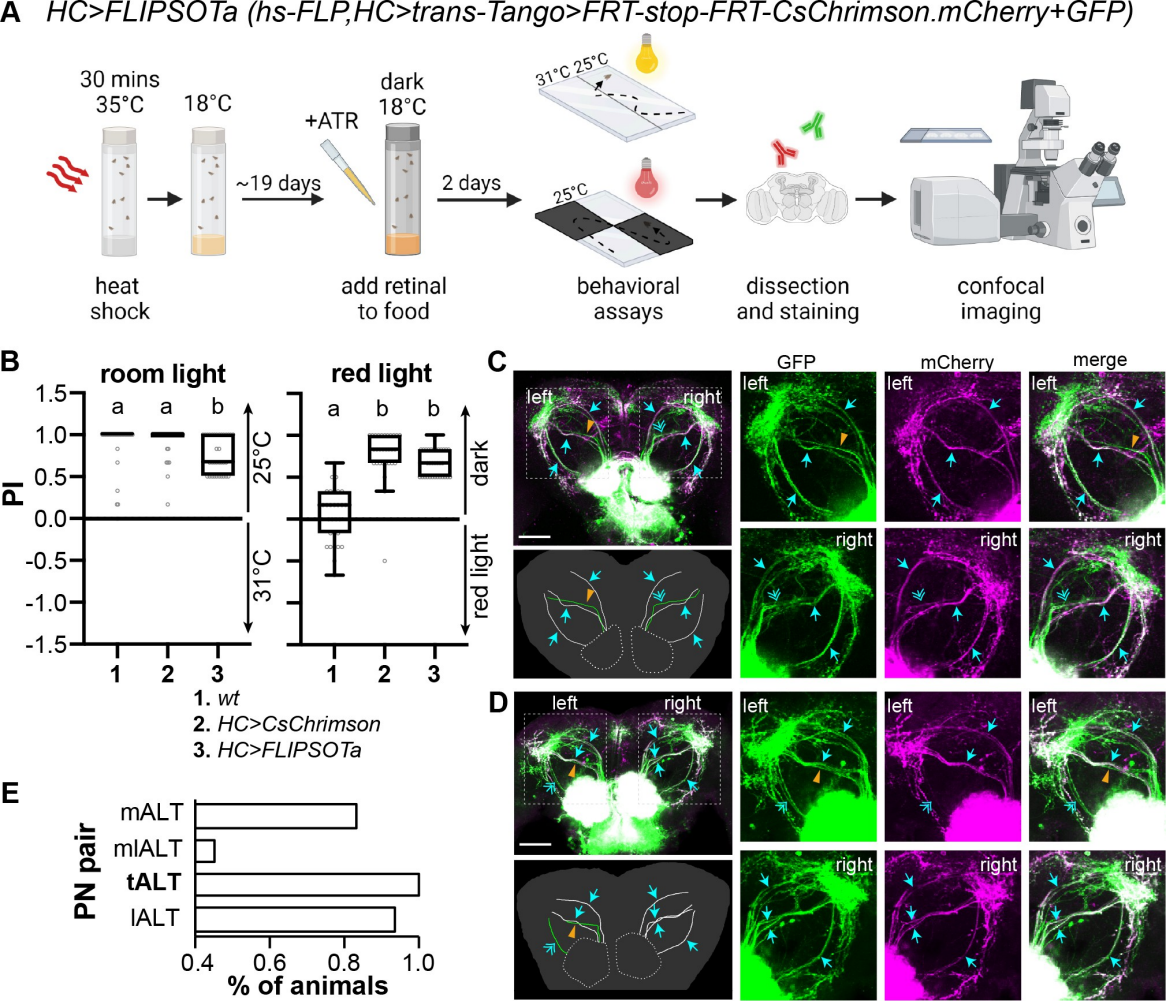
Identify PNs sufficient to drive avoidance. (A) Schematic representation of identifying PNs sufficient for the avoidance behavior. FLISOTa is *hs-FLP;UAS-trans-Tango/ QUAS-mCD8-GFP;QUAS-FRT-stop-FRT-CsChrimson*.*mCherry*. The pre-synaptic driver is *HC-Gal4* (created with BioRender.com). (B) PI of the indicated genotypes to avoid 31°C (left) or red light (right). Flies were raised with dietary retinal (ATR) in the dark for two days before experiments. *n* = 29–45; Kruskal-Wallis test followed by Dunn’s multiple comparisons test; letters denote statistically distinct groups, *p* < 0.05. The behavioral datasets of *wt* and *HC>CsChrimson* (*HC-Gal4*, *UAS-CsChrimson*.*mVenus)* from Fig 1B and 1F are included for comparison with *HC>FLIPSOTa* flies. (C,D) CsChrimson.mCherry-positive HC PNs in flies avoiding red light. Left upper panels: immunohistochemistry. Green: GFP antibody to show all postsynaptic neurons of HCs, magenta: mCherry antibody to indicate CsChrimson.mCherry-positive postsynaptic neurons of HCs; cyan arrow: CsChrimson.mCherry-positive PN, orange arrowhead: CsChrimson.mCherry-negative PN, cyan double arrow: CsChrimson.mCherry-weak PN; if the mCherry staining is much weaker than the GFP staining, the PN is considered to lack CsChrimson.mCherry. Scale bar: 50 μm. Left lower panels: illustration of PNs. White: CsChrimson.mCherry-positive PN, green: CsChrimson.mCherry-negative PN. Right three upper panels: left PNs; right three lower panels: right PNs. (C) Six PNs express CsChrimson.mCherry (both mALTs, tALTs, and lALT). (D) Six PNs express CsChrimson.mCherry (left: mALT and tALT; right: mALT, mlALT, tALT, and lALT). (E) The tALT is sufficient for the avoidance behavior. We analyzed 29 brains of *HC>FLIPSOTa*, all of which had at least one tALT expressing CsChrimson.mCherry. See also S5 and S6 Figs.

Then, we crossed *HC-Gal4* with *FLIPSOTa* to identify the individual PNs sufficient for rapid warm avoidance (Fig 6A). To achieve the optimal expression of *QUAS-FRT-stop-FRT-CsChrimson*.*mCherry*, we gave flies 21 days to develop *trans-*Tango. We conducted the single-fly rapid warm avoidance assay and tested 192 flies with normal warm avoidance. A slight but significant decrease in preference to room temperature observed in *HC>FLIPSOTa* was due to the fly age (S5B Fig). Then, the red light avoidance assay was performed, and 29 flies with strong red light avoidance were collected for brain dissection (Fig 6B). In *FLIPSOTa*, *QUAS-mCD8-GFP* served as a control to visualize postsynaptic neurons. Four tracts of PNs were observed on each hemisphere (green). In Fig 6C, the left mlALT did not express CsChrimson.mCherry (magenta), while the right mlALT only showed faint CsChrimson.mCherry. In Fig 6D, the left mlALT was absent of CsChrimson.mCherry, and the lALT had faint CsChrimson.mCherry. In the right brain hemisphere, all four tracts expressed CsChrimson.mCherry. All 29 brains of *HC>FLIPSOTa* had at least one tALT expressing CsChrimson.mCherry (Figs 6E and S6), indicating that the tALT is sufficient for driving rapid warm avoidance.

### *hs-FLP* causes leaky expression

When testing FLIPSOT, we noticed that even without heat shock, FLIPSOT results in fluorescence signals in the ALs. In some brains, PNs also have fluorescence signals. This leaky expression makes FLIPSOT unable to differentiate LNs. The expression of optogenetic genes in the AL may also indirectly impact behavior and weaken the argument of the behavioral function of PNs. Since each PN tract usually contains multiple PNs and the arborization of PNs in the AL and their target regions is a critical criterion for identifying different PNs in the same tract, the leaky expression also limits our ability to distinguish different PNs within the same PN tracts. Therefore, the immunostaining results from flies that do not pass relevant behavioral tests cannot provide useful information to identify the necessary neuronal tracts, as certain tract(s) may appear due to neurons that are in fact not necessary or sufficient. The strong signals without heat shock may result from *trans-*Tango [28] or bypassing the stop sequence in the FRT-stop-FRT cassette. These possibilities were ruled out, as the leaky expression was undetectable upon removal of *hs-FLP* from the system (S7 Fig). Another potential source of the leaky expression is trace amounts of FLP. Although the leaky expression is not observed in larval *Ir21a-QF2w* positive cells (Fig 4A and 4C), the long-period incubation for developing *trans-*Tango (10–21 days) may accumulate FLP, resulting in its expression sufficient to remove the stop sequence from the FRT-stop-FRT cassette in some cells. Raising flies in lower temperatures or using a different *hs-FLP* line may limit the FLP amount. When FLIPSOT is applied to analyze PNs, users may find it beneficial to draw conclusions based on flies that pass the behavioral assays and entirely lack specific tracts in both hemispheres. Complete absence implies that the tract does not contain sufficient or necessary PNs and, importantly, avoids the potential complications of leaky expression.

## Discussion

FLIPSOT relies on a presynaptic driver to identify the behavioral function of individual postsynaptic neurons. FLIPSOT uses (1) *trans-*Tango to access postsynaptic neurons genetically, (2) optogenetic approaches to activate (FLIPSOTa) or inhibite (FLIPSOTi) postsynaptic neurons in a random and sparse manner, and (3) fluorescence markers tagged with optogenetic genes to visualize these neurons. As long as a single-fly behavioral assay is available to reflect the function of presynaptic neurons, FLIPSOT is readily applied to identify functions of individual postsynaptic neurons in many other fruit fly neural circuits. It also has the potential to be adapted for use in mammalian circuits.

FLIPSOT was created to provide a more comprehensive understanding of the behavioral functions of postsynaptic neurons. Many methods depend on the similarity in morphology to identify postsynaptic drivers [41,50,53]. However, the same neuron may have different morphologies in different flies, even between two sides of a single brain. For example, two mlALTs in Fig 3A exhibit different morphologies. Moreover, when the neurons are packed heavily, and multiple neurons have similar morphologies, relying on morphology to determine postsynaptic drivers becomes impracticable, and validation of direct synaptic interaction is not trivial. Therefore, morphology-dependent approaches cannot comprehensively investigate the function of postsynaptic neurons. FLIPSOT adopts *trans-*Tango, which provides genetic access to postsynaptic neurons in a more comprehensive manner using a presynaptic driver. For example, *HC>trans-Tango* labels all four tracts of HC postsynaptic neurons identified by EM tracing [50] (Fig 3A and 3B). Among these four tracts, FLIPSOTi demonstrates that the mALT is necessary to drive rapid warm avoidance (Figs 5 and S6), consistent with a previous report [17]. The tALT is a novel PN with unknown functions [50]. FLIPSOTa suggests that activation of the tALT is sufficient to drive avoidance (Figs 6 and S6). In addition, FLIPSOT has the potential to quantitively assess neural information transmission by establishing a correlation between the behavioral outputs and brain staining.

When implementing FLIPSOT, the following suggestions may help efficiently achieve optimal outcomes. (1) In FLIPSOT, *trans*-Tango controls an optogenetic tool with a fluorescence marker and a reporter. This reporter labels all postsynaptic neurons and thus serves as an internal control to determine the neurons expressing optogenetic genes. Even though both reporters and fluorescence markers can be observed under confocal microscopy, we suggest using immunohistochemistry to enhance their signals and limit some false-positive noise. (2) The stochasticity of FLISPOT leads to the possibility that FLIPSOT is expressed in one set of neurons in one hemisphere and is expressed in a different set of neurons in the other hemisphere. This randomness precludes from arriving at conclusions until a particular set is concomitantly expressed FLIPSOT. If removing neural circuits from one hemisphere is easy to achieve and does not impact flies’ behavior, we suggest doing so before testing the behavioral output. This manipulation may provide an efficient way to reach a conclusion. Since single-side PNs are sufficient for guiding warm avoidance behavior, we tested the feasibility of this idea (S8 Fig). This *HC>FLIPSOTa* fly with the right arista removed avoided the red light. Immunostaining showed three PNs expressed CsChrimson.mCherry (mALTs, tALTs, and lALT) in the left hemisphere, suggesting the mlALT tract is insufficient for driving avoidance.

In the future, besides solving the leaky expression problem, we will generate a new FLIPSOT variant expressing both inhibitory and activatable optogenetic tools to test necessity and sufficiency in the same flies. Expression of both GtACR2 and CsChrimson in HCs leads to inhibition of HCs by blue light and activation of HCs in red light (S9 Fig). Moreover, the combination of a genetic calcium indicator and optogenetic tools may help understand the physiological responses of the individual postsynaptic neurons in response to environmental stimuli.

## Materials and methods

### Drosophila strains

*w*^*1118*^ was used as a genetic background control for all behavioral assays. *UAS-GtACR2* [44], *HC-Gal4* [41], *Gr28b*.*d-Gal4* [54], *UAS-CsChrimson* [42] (RRID:BDSC_55135 and 55136), *hs-FLP* [55] (RRID:BDSC_6), *UAS-FRT-stop-FRT-GFP* [56] (RRID:BDSC_30032), *QUAS-mCD8-GFP* [57] (RRID:BDSC_30002), *QUAS-mtdTomato-HA* [57] (RRID:BDSC_30004), *QUAS-GCaMP3* (RRID:BDSC_65684), and *UAS-trans-Tango* [28] (RRID:BDSC_77123 and 77124) were previously described.

To create the *QUAS-FRT-stop-FRT-CsChrimson*.*mCherry* and *QUAS-FRT-stop-FRT-GtACR2*.*EYFP* lines, the *FRT-stop-FRT* sequence [46] (Addgene plasmid #64716) was cloned into the *pQUAST-attB* (RRID:DGRC_1438) vector. Then, either the *CsChrimson*.*mCherry* [42] (Addgene plasmid #111547) sequence or the *GtACR2*.*EYFP* [45] (Addgene plasmid #67877) sequence was cloned into the vector. The final constructs were integrated into attP2 [58] (RRID:DGRC_8622). *QUAS-FRT-stop-FRT-CsChrimson*.*mCherry* and *QUAS-FRT-stop-FRT-GtACR2*.*EYFP* were then crossed with *QUAS-mCD8-GFP* and *QUAS-mtdTomato-HA*, respectively, to create *QUAS-mCD8-GFP;QUAS-FRT-stop-FRT-CsChrimson*.*mCherry* and *QUAS-mtdTomato-HA;QUAS-FRT-stop-FRT-GtACR2*.*EYFP*.

*Ir21a-QF2w* was created by removing the *synaptobrevin* promoter and subcloning the *Ir21a* promoter region into *pattB-synaptobrevin-QF2w-hsp70* [59,60] (Addgene plasmid #46116). The construct was also integrated into attP40 [58] (RRID:DGRC_8622).

### Single-fly rapid warm avoidance assay and optogenetic assay

Flies were maintained at 22°C (or 18°C for *trans-*Tango development) in cornmeal medium. The light:dark cycle mimicked the natural sunrise and sunset time. 1 L cornmeal medium contained 1 L dH_2_O, 79 g dextrose, 7.5 g agar, 24 g flaked yeast, 57 g cornmeal, 2.1 g methal-4-hydroxybenzoate (dissolved in 11.1 mL ethanol), 6 g sodium potassium tartrate tetrahydrate, and 0.9 g calcium chloride. All behavioral experiments were performed between 12:00 pm and 7:00 pm. Two days before behavioral experiments, flies were placed in the dark on food supplemented 40 μM ATR (Sigma-Aldrich).

The rapid warm avoidance assay was performed in a behavioral room, in which the temperature was set at 25°C. Two steel plates were used to set up the temperature gradience. One steel plate was placed on top of a hot plate, and the other one was lifted to align with the first steel plate. A sheet protector was taped on top. The hot plate temperature was adjusted so that the surface of the steel plate was about 31°C. Before this assay, a custom-made fly pooter was made for each operator. The fly pooter was made of a thin plastic tube and two 3mL transfer pipettes. The bulbs of the transfer pipettes were removed, and the tips were attached to each end of the plastic tube with a small piece of insect netting. A single fly was placed under a plastic cover (2 mm (height) X 58 mm (width) X 83 mm (length)) using a fly pooter and allowed to acclimate for 15–25 seconds. Flies were moved to 25°C to start the behavior. The position of the fly was recorded every five seconds for one minute. Flies that did not move for over 30 seconds were discarded. A desk lamp (room light) (~22 lux) was used to illuminate the rapid warm avoidance assay. A blue light source (~20 klux) was built [61] to activate GtACR2. The preference index (PI) was calculated using the following formula:

$$PI=\frac{\left(time\;point\;number\;in\;25{}^{\circ}C\;)-(time\;point\;number\;in\;31{}^{\circ}C\;\right)}{total\;time\;point\;number}$$

Red light optogenetic assays were conducted at 25°C. The base of the experiment was a sheet protector overlaid on a black background. Flies were placed on the sheet protector under a plastic cover with two opposing transparent quadrants and two opaque quadrants. The red light illuminated the cover from the top. Flies were given ten seconds to acclimate, and all experiments started with flies in one of the visible quadrants. Flies that did not move for more than 30 seconds were discarded. The red light source (32 klux) was previously described [61]. The preference index (PI) was calculated using the following formula:

$$PI=\frac{\left(time\;point\;number\;in\;the"dark")-(time\;point\;number\;in\;red\;light\;\right)}{total\;time\;point\;number}$$

### Brain dissection and immunostaining

Brains were dissected using forceps (FST 11413–11) in 1X PBS and then transferred to PBS on ice. Immunohistochemistry was performed as previously described with some modifications [61]. Brains were fixed in 1X PBS with 4% paraformaldehyde for 30 min. After washing three times (5 min each) in PBST (1X PBS with 0.5% Triton-100), antigen retrieval was performed by incubating brains in 1X PBS with 0.1% SDS and 20% H_2_O_2_ for 30 min. Next, brains were washed three times in PBST (5 min each), blocked in 10% NGS for one hour, and incubated in the primary antibody solution for three days. After washing overnight in PBST, brains were transferred to the secondary antibody solution overnight and then washed in PBST overnight. Samples were mounted using Vectashield Mounting Medium (VECTASHIELD) and imaged using a Nikon A1 Confocal Microscope. Primary antibodies used were rabbit anti-dsRed (1:200, Takara Bio), rabbit anti-GFP (1:500, Invitrogen), mouse anti-GFP (1:500, Sigma-Aldrich), and mouse anti-HA (1:200, Invitrogen). Secondary antibodies included goat anti-rabbit Cy3 (1:250, Jackson Immuno), goat anti-rabbit Alexa Fluor 488 (1:250, Jackson Immuno), goat anti-mouse CF488A (1:250, Sigma-Aldrich), and goat anti-mouse TRITC (1:250, Invitrogen).

### Statistical analysis

Statistical details of experiments were mentioned in the figure legends. The normality of distributions was assessed by the Shapiro-Wilk W test (*p* ≤ 0.05 rejected normal distribution). Statistical comparisons were performed by the Mann-Whitney test or Kruskal-Wallis test. Data analysis was conducted using GraphPad Prism 9. Schematic diagrams of Figs 1, 2A, 2C, 2E, 5A, and 6A were created with BioRender.com.

## Supporting information

S1 Fig PI is not affected by fly gender, dietary retinal (ATR), or whether 31°C is on the left or right. (A) PI of the indicated genders that were raised with (+) or without (-) dietary retinal (ATR). *n* = 14–28; Kruskal-Wallis test followed by Dunn’s multiple comparisons test. (B) PI from tests that 31°C was on the left, on the right or both sides were 25°C. *n* = 15; data represent means ± SEM; Kruskal-Wallis test followed by Dunn’s multiple comparisons test; ****, *p* < 0.0001.
(PDF)

S2 Fig *Gr28b*.*d-Gal4* drives *trans-*Tango to label postsynaptic neurons of HCs. (A) In flies bearing *trans*-Tango components, driving ligands and GFP expressing in VP2 (black arrow) of the AL by *Gr28b*.*d-Gal4* results in mtdTomato expression (magenta) in postsynaptic LNs and PNs (cyan arrow). Scale bar: 50 μm. (B) Illustration of PNs. (C) Left PNs. (D) Right PNs.
(PDF)

S3 Fig *Ir21a-QF2w* labels three neurons in each DOG. Genotype: *Ir21a-QF2w/QUAS-GCaMP3*. White arrow: cell body. Scale bar: 10 μm.
(PDF)

S4 Fig Heat shock removes the stop sequence from the FRT-stop-FRT cassette in HCs randomly. Among 61 aristae observed, ten had zero HCs (A), eight had one HC (B), 23 had two HCs (C), and 20 had three HCs (D). Scale bar: 10 μm.
(PDF)

S5 Fig The rapid warm avoidance of old flies. (A) PI of the indicated genotypes and ages. *n* = 15–34; Mann-Whitney test comparing to *wt* (2-7d) or the indicated group; **, *p* < 0.01; ns, not significant. The behavioral datasets of *wt* (2-7d) and *HC>FLIPSOTi* (~10d) are the same as in Fig 5B. (B) PI of the indicated genotypes and ages. *n* = 17–34; Mann-Whitney test comparing to *wt* (2-7d) or the indicated group; *, *p* < 0.05; **, *p* < 0.01; ns, not significant. The behavioral datasets of *wt* (2-7d) and *HC>FLIPSOTa* (~21d) are the same as in Fig 6B.
(PDF)

S6 Fig The mALT and tALT VP2 PNs identified from the full adult fly brain EM tracing. The mALT tract (green) is necessary to drive avoidance to high temperatures, while the tALT tract (magenta) is sufficient for the avoidance behavior. (A) The mALT and tALT PNs are displayed with the brain structure. (B) The brain structure is removed.
(PDF)

S7 Fig Removal of *hs-FLP* eliminates the leaky expression. (A) The expression of GtACR2.EYFP and mtdTomato in *HC-Gal4*,*UAS-trans-Tango/QUAS-mtdTomato;QUAS-FRT-stop-FRT-GtACR2*.*EYFP*. Cyan arrows: four tracts of postsynaptic PNs labeled by mtdTomato. Scale bar: 100 μm. (B) The expression of CsChrimson.mCherry and GFP in *HC-Gal4*,*UAS-trans-Tango/QUAS-mCD-GFP;QUAS-FRT-stop-FRT-CsChrimson*.*mCherry*. Cyan arrows: four tracts of postsynaptic PNs labeled by GFP. Scale bar: 100 μm.
(PDF)

S8 Fig CsChrimson.mCherry-positive HC PNs in a *HC>FLIPSOTa* fly avoiding red light with the ablation of the right arista. Three PNs expressed CsChrimson.mCherry (mALTs, tALTs, and lALT) in the left hemisphere. The PI value of the single-fly two-choice thermotactic assay under ambient light condition was 1.00, and the PI value of the single-fly optogenetic assay under red light condition was 0.67. Green: GFP antibody to show all postsynaptic neurons of HCs in the left hemisphere, magenta: mCherry antibody to indicate CsChrimson.mCherry-positive postsynaptic neurons of HCs; cyan arrow: CsChrimson.mCherry-positive PN, orange arrowhead: CsChrimson.mCherry-negative PN, cyan double arrow: CsChrimson.mCherry-weak PN. Scale bar: 100 μm.
(PDF)

S9 Fig Single-fly thermotactic and optogenetic assays of *HC>GtACR2/CsChrimson*. (A) PI of *HC>GtACR2/CsChrimson* that were raised with (+) or without (-) dietary retinal (ATR) under ambient room light condition. *n* = 30; Mann-Whitney test; **, *p* < 0.01. *HC>GtACR2/CsChrimson* is *HC-Gal4;UAS-GtACR2*.*EYFP/UAS-CsChrimson*.*mVenus*. (B) PI of *HC>GtACR2/CsChrimson* that were raised with (+) or without (-) dietary retinal (ATR) under blue light condition. *n* = 30; Mann-Whitney test; ****, *p* < 0.0001. (C) PI of *HC>GtACR2/CsChrimson* that were raised with (+) or without (-) dietary retinal (ATR) under red light condition. *n* = 30; Mann-Whitney test; ****, *p* < 0.0001.
(PDF)
