# Supplementary material for: Functional labeling of individualized postsynaptic neurons using optogenetics and trans-Tango in Drosophila (FLIPSOT)
Source: PLoS Genet. 2024 Mar 14;20(3):e1011190. doi: 10.1371/journal.pgen.1011190 (PMC10965055; doi:10.1371/journal.pgen.1011190)
Supplement: S1 Fig — (A) PI of the indicated genders that were raised with (+) or without (-) dietary retinal (ATR). n = 14–28; Kruskal-Wallis test followed by Dunn’s multiple comparisons test. (B) PI from tests that 31°C was on the left, on the right or both sides were 25°C. n = 15; data represent means ± SEM; Kruskal-Wallis test followed by Dunn’s multiple comparisons test; ****, p < 0.0001. (PDF) [file pgen.1011190.s001.pdf]

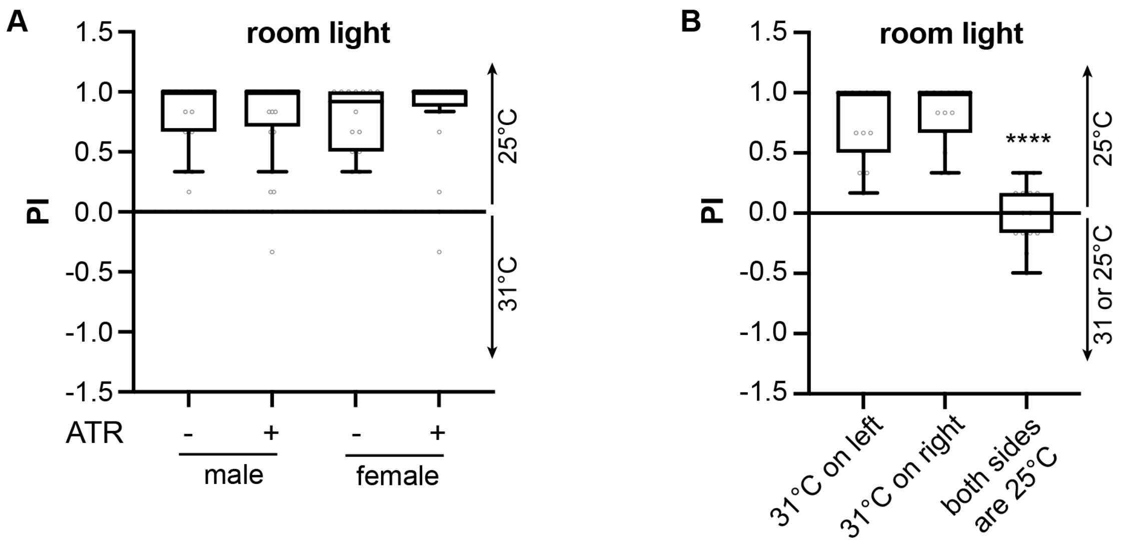

S1 Fig. PI is not affected by fly gender, dietary retinal (ATR), or whether 31°C is on the left or right.

(A) PI of the indicated genders that were raised with (+) or without (-) dietary retinal (ATR). n = 14-28; Kruskal-Wallis test followed by Dunn's multiple comparisons test.

(B) PI from tests that 31°C was on the left, on the right or both sides were 25°C. n = 15; data represent means ± SEM; Kruskal-Wallis test followed by Dunn's multiple comparisons test; ****, $p < 0.0001$.
