# Supplementary material for: Functional labeling of individualized postsynaptic neurons using optogenetics and trans-Tango in Drosophila (FLIPSOT)
Source: PLoS Genet. 2024 Mar 14;20(3):e1011190. doi: 10.1371/journal.pgen.1011190 (PMC10965055; doi:10.1371/journal.pgen.1011190)
Supplement: S2 Fig — (A) In flies bearing trans-Tango components, driving ligands and GFP expressing in VP2 (black arrow) of the AL by Gr28b.d-Gal4 results in mtdTomato expression (magenta) in postsynaptic LNs and PNs (cyan arrow). Scale bar: 50 μm. (B) Illustration of PNs. (C) Left PNs. (D) Right PNs. (PDF) [file pgen.1011190.s002.pdf]

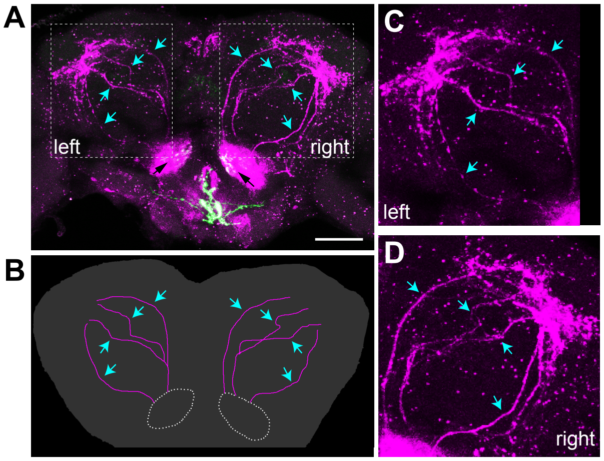

S2 Fig. *Gr28b.d-Gal4* drives *trans*-Tango to label postsynaptic neurons of HCs.

(A) In flies bearing *trans*-Tango components, driving ligands and GFP expressing in VP2 (black arrow) of the AL by *Gr28b.d-Gal4* results in mtdTomato expression (magenta) in postsynaptic LNs and PNs (cyan arrow). Scale bar: 50 μm.

(B) Illustration of PNs.

(C) Left PNs.

(D) Right PNs.
