# Supplementary material for: Functional labeling of individualized postsynaptic neurons using optogenetics and trans-Tango in Drosophila (FLIPSOT)
Source: PLoS Genet. 2024 Mar 14;20(3):e1011190. doi: 10.1371/journal.pgen.1011190 (PMC10965055; doi:10.1371/journal.pgen.1011190)
Supplement: S3 Fig — Genotype: Ir21a-QF2w/QUAS-GCaMP3. White arrow: cell body. Scale bar: 10 μm. (PDF) [file pgen.1011190.s003.pdf]

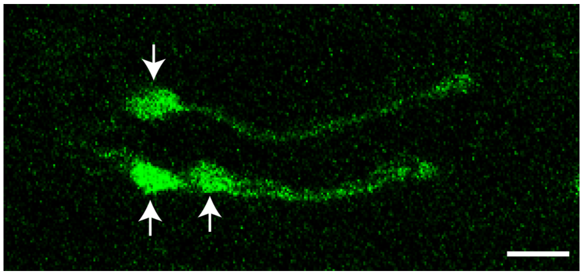

S3 Fig. *Ir21a-QF2w* labels three neurons in each DOG. Genotype: *Ir21a-QF2w/QUAS-GCaMP3*. White arrow: cell body. Scale bar: 10 μm.
