# Supplementary material for: Functional labeling of individualized postsynaptic neurons using optogenetics and trans-Tango in Drosophila (FLIPSOT)
Source: PLoS Genet. 2024 Mar 14;20(3):e1011190. doi: 10.1371/journal.pgen.1011190 (PMC10965055; doi:10.1371/journal.pgen.1011190)
Supplement: S4 Fig — Among 61 aristae observed, ten had zero HCs (A), eight had one HC (B), 23 had two HCs (C), and 20 had three HCs (D). Scale bar: 10 μm. (PDF) [file pgen.1011190.s004.pdf]

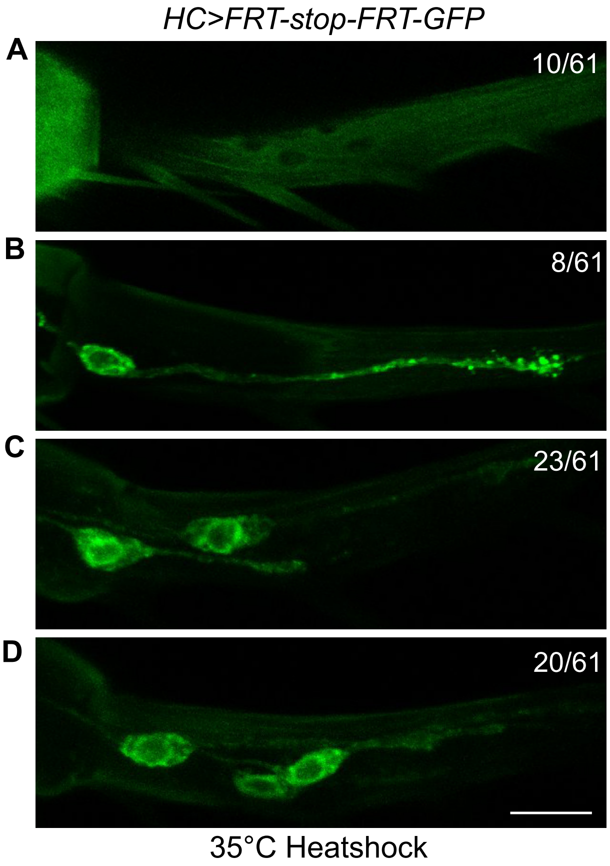

S4 Fig. Heat shock removes the stop sequence from the FRT-stop-FRT cassette in HCs randomly. Among 61 aristae observed, ten had zero HCs (A), eight had one HC (B), 23 had two HCs (C), and 20 had three HCs (D). Scale bar: 10 μm.
