# Supplementary material for: Functional labeling of individualized postsynaptic neurons using optogenetics and trans-Tango in Drosophila (FLIPSOT)
Source: PLoS Genet. 2024 Mar 14;20(3):e1011190. doi: 10.1371/journal.pgen.1011190 (PMC10965055; doi:10.1371/journal.pgen.1011190)
Supplement: S5 Fig — (A) PI of the indicated genotypes and ages. n = 15–34; Mann-Whitney test comparing to wt (2-7d) or the indicated group; **, p < 0.01; ns, not significant. The behavioral datasets of wt (2-7d) and HC>FLIPSOTi (~10d) are the same as in Fig 5B. (B) PI of the indicated genotypes and ages. n = 17–34; Mann-Whitney test comparing to wt (2-7d) or the indicated group; *, p < 0.05; **, p < 0.01; ns, not significant. The behavioral datasets of wt (2-7d) and HC>FLIPSOTa (~21d) are the same as in Fig 6B. (PDF) [file pgen.1011190.s005.pdf]

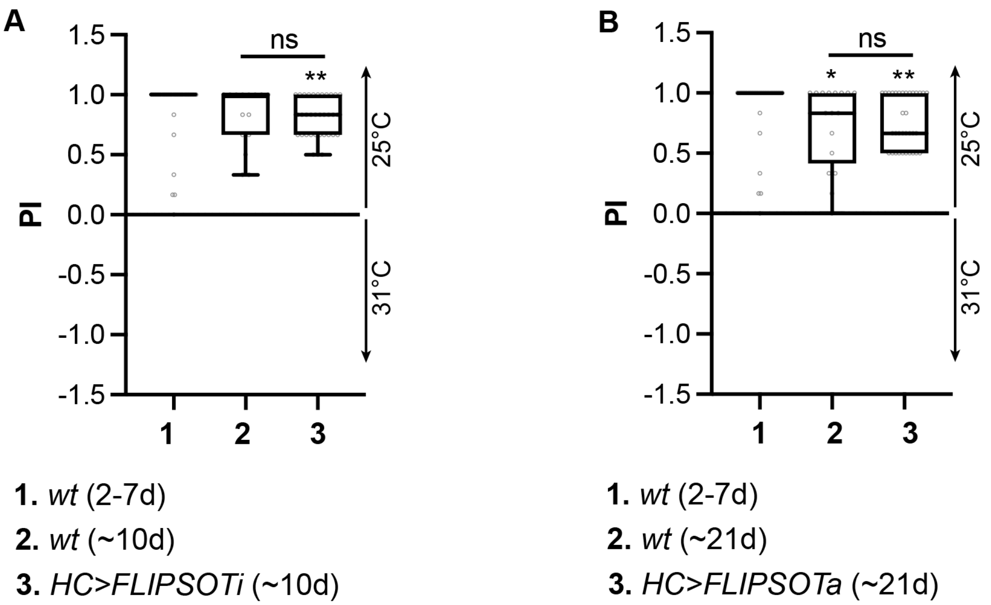

S5 Fig. The rapid warm avoidance of old flies.

(A) PI of the indicated genotypes and ages. n = 15-34; Mann-Whitney test comparing to *wt* (2-7d) or the indicated group; **, $p < 0.01$; ns, not significant. The behavioral datasets of *wt* (2-7d) and *HC>FLIPSOTi* (~10d) are the same as in Fig 5B.

(B) PI of the indicated genotypes and ages. n = 17-34; Mann-Whitney test comparing to *wt* (2-7d) or the indicated group; *, $p < 0.05$; **, $p < 0.01$; ns, not significant. The behavioral datasets of *wt* (2-7d) and *HC>FLIPSOTa* (~21d) are the same as in Fig 6B.
