# Supplementary material for: Functional labeling of individualized postsynaptic neurons using optogenetics and trans-Tango in Drosophila (FLIPSOT)
Source: PLoS Genet. 2024 Mar 14;20(3):e1011190. doi: 10.1371/journal.pgen.1011190 (PMC10965055; doi:10.1371/journal.pgen.1011190)
Supplement: S6 Fig — The mALT tract (green) is necessary to drive avoidance to high temperatures, while the tALT tract (magenta) is sufficient for the avoidance behavior. (A) The mALT and tALT PNs are displayed with the brain structure. (B) The brain structure is removed. (PDF) [file pgen.1011190.s006.pdf]

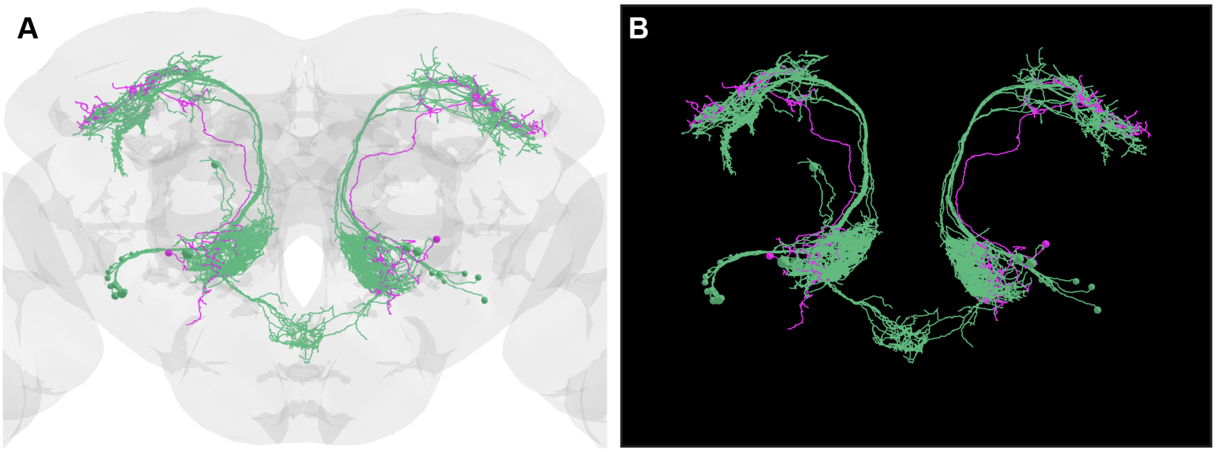

S6 Fig. The mALT and tALT VP2 PNs identified from the full adult fly brain EM tracing. The mALT tract (green) is necessary to drive avoidance to high temperatures, while the tALT tract (magenta) is sufficient for the avoidance behavior. (A) The mALT and tALT PNs are displayed with the brain structure. (B) The brain structure is removed.
