# Supplementary material for: Functional labeling of individualized postsynaptic neurons using optogenetics and trans-Tango in Drosophila (FLIPSOT)
Source: PLoS Genet. 2024 Mar 14;20(3):e1011190. doi: 10.1371/journal.pgen.1011190 (PMC10965055; doi:10.1371/journal.pgen.1011190)
Supplement: S7 Fig — (A) The expression of GtACR2.EYFP and mtdTomato in HC-Gal4,UAS-trans-Tango/QUAS-mtdTomato;QUAS-FRT-stop-FRT-GtACR2.EYFP. Cyan arrows: four tracts of postsynaptic PNs labeled by mtdTomato. Scale bar: 100 μm. (B) The expression of CsChrimson.mCherry and GFP in HC-Gal4,UAS-trans-Tango/QUAS-mCD-GFP;QUAS-FRT-stop-FRT-CsChrimson.mCherry. Cyan arrows: four tracts of postsynaptic PNs labeled by GFP. Scale bar: 100 μm. (PDF) [file pgen.1011190.s007.pdf]

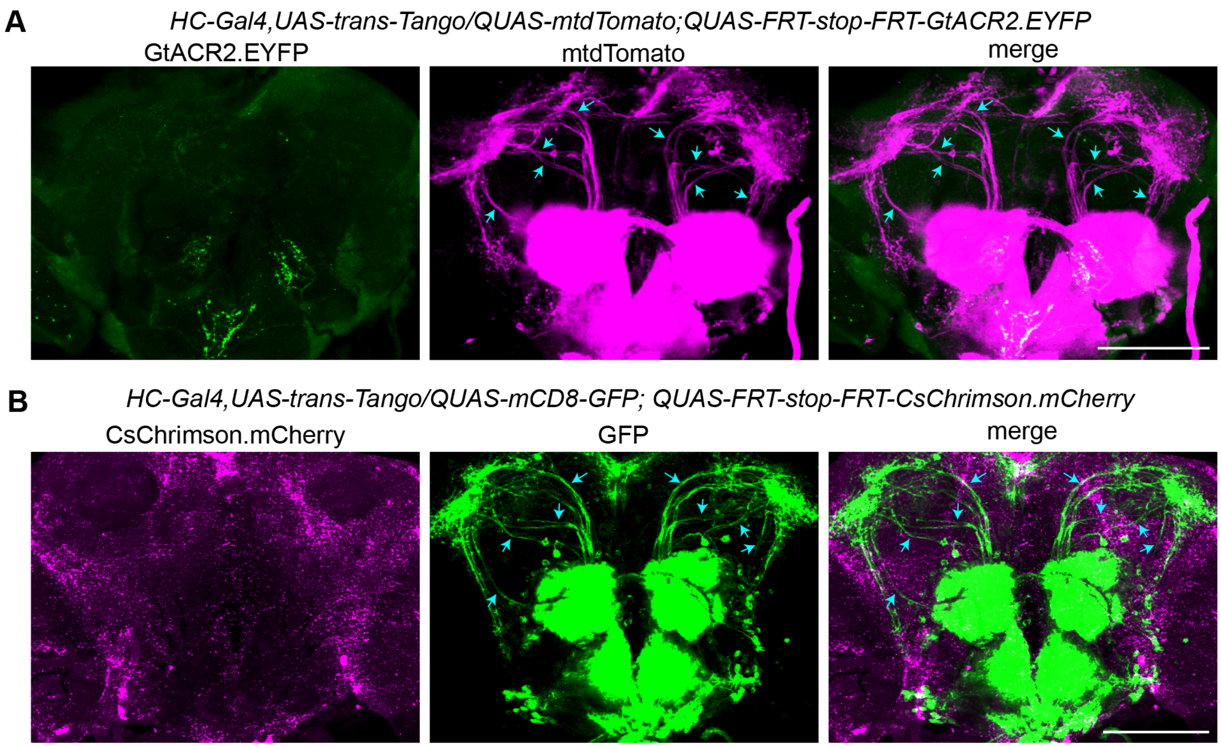

S7 Fig. Removal of *hs-FLP* eliminates the leaky expression.

(A) The expression of GtACR2.EYFP and mtdTomato in *HC-Gal4,UAS-trans-Tango/QUAS-mtdTomato;QUAS-FRT-stop-FRT-GtACR2.EYFP*. Cyan arrows: four tracts of postsynaptic PNs labeled by mtdTomato. Scale bar: 100 μm.

(B) The expression of CsChrimson.mCherry and GFP in *HC-Gal4,UAS-trans-Tango/QUAS-mCD-GFP;QUAS-FRT-stop-FRT-CsChrimson.mCherry*. Cyan arrows: four tracts of postsynaptic PNs labeled by GFP. Scale bar: 100 μm.
