# Supplementary material for: Functional labeling of individualized postsynaptic neurons using optogenetics and trans-Tango in Drosophila (FLIPSOT)
Source: PLoS Genet. 2024 Mar 14;20(3):e1011190. doi: 10.1371/journal.pgen.1011190 (PMC10965055; doi:10.1371/journal.pgen.1011190)
Supplement: S8 Fig — Three PNs expressed CsChrimson.mCherry (mALTs, tALTs, and lALT) in the left hemisphere. The PI value of the single-fly two-choice thermotactic assay under ambient light condition was 1.00, and the PI value of the single-fly optogenetic assay under red light condition was 0.67. Green: GFP antibody to show all postsynaptic neurons of HCs in the left hemisphere, magenta: mCherry antibody to indicate CsChrimson.mCherry-positive postsynaptic neurons of HCs; cyan arrow: CsChrimson.mCherry-positive PN, orange arrowhead: CsChrimson.mCherry-negative PN, cyan double arrow: CsChrimson.mCherry-weak PN. Scale bar: 100 μm. (PDF) [file pgen.1011190.s008.pdf]

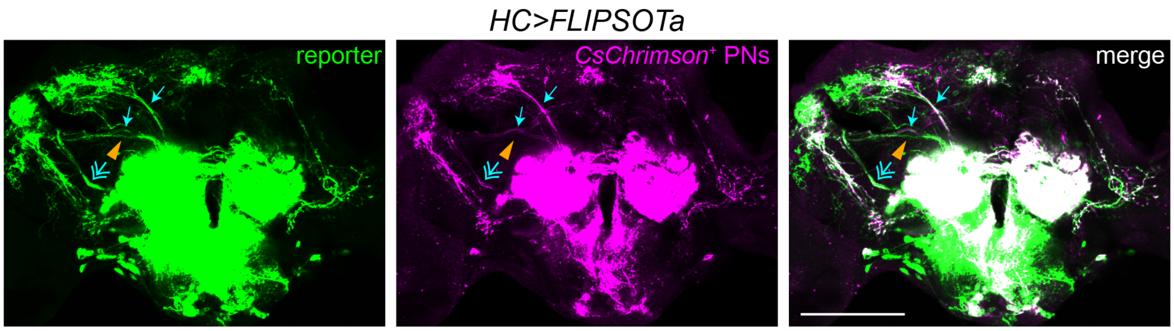

S8 Fig. CsChrimson.mCherry-positive HC PNs in a *HC>FLIPSOTa* fly avoiding red light with the ablation of the right arista. Three PNs expressed CsChrimson.mCherry (mALTs, tALTs, and lALT) in the left hemisphere. The PI value of the single-fly two-choice thermotactic assay under ambient light condition was 1.00, and the PI value of the single-fly optogenetic assay under red light condition was 0.67. Green: GFP antibody to show all postsynaptic neurons of HCs in the left hemisphere, magenta: mCherry antibody to indicate CsChrimson.mCherry-positive postsynaptic neurons of HCs; cyan arrow: CsChrimson.mCherry-positive PN, orange arrowhead: CsChrimson.mCherry-negative PN, cyan double arrow: CsChrimson.mCherry-weak PN. Scale bar: 100 μm.
