# Supplementary material for: Functional labeling of individualized postsynaptic neurons using optogenetics and trans-Tango in Drosophila (FLIPSOT)
Source: PLoS Genet. 2024 Mar 14;20(3):e1011190. doi: 10.1371/journal.pgen.1011190 (PMC10965055; doi:10.1371/journal.pgen.1011190)
Supplement: S9 Fig — (A) PI of HC>GtACR2/CsChrimson that were raised with (+) or without (-) dietary retinal (ATR) under ambient room light condition. n = 30; Mann-Whitney test; **, p < 0.01. HC>GtACR2/CsChrimson is HC-Gal4;UAS-GtACR2.EYFP/UAS-CsChrimson.mVenus. (B) PI of HC>GtACR2/CsChrimson that were raised with (+) or without (-) dietary retinal (ATR) under blue light condition. n = 30; Mann-Whitney test; ****, p < 0.0001. (C) PI of HC>GtACR2/CsChrimson that were raised with (+) or without (-) dietary retinal (ATR) under red light condition. n = 30; Mann-Whitney test; ****, p < 0.0001. (PDF) [file pgen.1011190.s009.pdf]

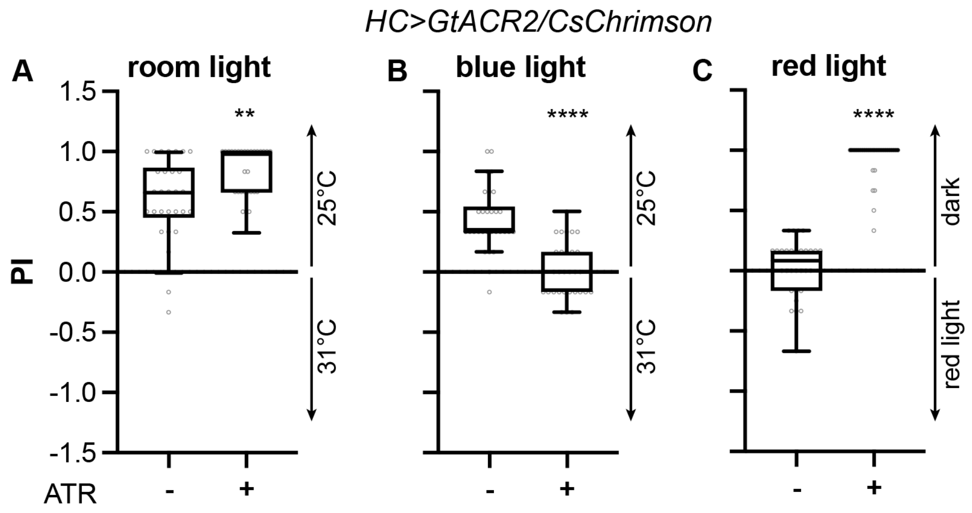

S9 Fig. Single-fly thermotactic and optogenetic assays of *HC>GtACR2/CsChrimson*.
(A) PI of *HC>GtACR2/CsChrimson* that were raised with (+) or without (-) dietary retinal (ATR) under ambient room light condition. $n = 30$; Mann-Whitney test; **, $p < 0.01$. *HC>GtACR2/CsChrimson* is *HC-Gal4;UAS-GtACR2.EYFP/UAS-CsChrimson.mVenus.*
(B) PI of *HC>GtACR2/CsChrimson* that were raised with (+) or without (-) dietary retinal (ATR) under blue light condition. $n = 30$; Mann-Whitney test; ****, $p < 0.0001$.
(C) PI of *HC>GtACR2/CsChrimson* that were raised with (+) or without (-) dietary retinal (ATR) under red light condition. $n = 30$; Mann-Whitney test; ****, $p < 0.0001$.
